# Elevated Serum Cyclophilin B Levels Are Associated with the Prevalence and Severity of Metabolic Syndrome

**DOI:** 10.3389/fendo.2017.00360

**Published:** 2017-12-22

**Authors:** Hang Zhang, Qin Fan, Hongyang Xie, Lin Lu, Rong Tao, Fang Wang, Rui Xi, Jian Hu, Qiujing Chen, Weifeng Shen, Ruiyan Zhang, Xiaoxiang Yan

**Affiliations:** ^1^Department of Cardiology, Rui Jin Hospital, Shanghai Jiao Tong University School of Medicine, Shanghai, China; ^2^Institute of Cardiovascular Diseases, Shanghai Jiao Tong University School of Medicine, Shanghai, China

**Keywords:** cyclophilin B, CD147, metabolic syndrome, inflammation, cardiovascular risk

## Abstract

**Objective:**

Inflammation plays a central role in the pathogenesis of metabolic syndrome (MetS). Cyclophilin B (CypB) can be constitutively secreted in response to inflammatory stimuli and oxidative stress, participating in tissue or systemic inflammation. We investigated the relationship between CypB and MetS in both humans and mice.

**Methods:**

Serum CypB levels were determined in 211 subjects with MetS and 292 subjects without MetS (non-MetS) (133 healthy controls and 159 high-risk subjects with one to two MetS components). Additionally, CypB expression in metabolic organs was examined in mice fed with high-fat diet (HFD) and genetically obese (ob/ob) mice.

**Results:**

Serum CypB level was significantly higher in MetS subjects compared with both groups of non-MetS subjects (193.80 ± 83.22 vs. 168.38 ± 65.01 vs. 124.26 ± 47.83 ng/mL, *P* < 0.001). Particularly, serum CypB level was significantly higher in subjects with hypertension, central obesity, diabetes mellitus or hyperglycemia, elevated levels of triglycerides, or reduced levels of high-density lipoprotein than in those without. Moreover, CypB was positively associated with the number of MetS components (*r* = 0.404, *P* < 0.001), indicating that a higher serum CypB level reflected more severe MetS. Multivariate regression revealed that a one SD increase in CypB was associated with an odds ratio of 1.506 (1.080–2.101, *P* = 0.016) for MetS prevalence after adjusting for age, gender, conventional risk factors, and medication. Stratified analyses by age and gender demonstrated that subjects >60 years old with higher CypB levels were more likely to have MetS, and the risk for MetS was higher and more significant in women compared with men. Additionally, CypB expression levels were lower at baseline and dramatically enhanced in metabolic organs (such as the liver) and visceral and subcutaneous adipose tissue from HFD-induced obese mice and ob/ob mice.

**Conclusion:**

Increased CypB levels were significantly and independently associated with the presence and severity of MetS, indicating that CypB could be used as a novel biomarker and clinical predictor of MetS.

## Introduction

As a cluster of cardiovascular risk factors, metabolic syndrome (MetS) consists of multiple metabolic abnormalities including central obesity, impaired fasting blood glucose or insulin resistance, dyslipidemia, and hypertension (1). It has become a major public health problem worldwide, having a critical role in the origin of several cardiometabolic diseases such as cardiovascular disease and type 2 diabetes mellitus (DM) (1, 2) and being associated with an increased risk of developing certain types of cancer (3). In Chinese adults, the prevalence of MetS was 20–25% as reported by a national survey in 2013 (4). Accumulating evidence including ours has demonstrated that inflammation and immune response play an essential role in lipid metabolism, insulin resistance, obesity, and MetS development, and some related factors were found to be of vital importance with diagnostic and prognostic value or as therapeutic targets (5–7).

Cyclophilins are ubiquitously distributed intracellular proteins originally described as the host cell receptors for the immunosuppressive drug cyclosporin A (8). As a member of the cyclophilin family and mainly existing in the endoplasmic reticulum and the nucleus, intracellular cyclophilin B (CypB) acts as a molecular chaperone and exerts various functions due to its peptidyl-prolyl cis-trans isomerase activity, which contributes to protein folding, secretion, and posttranslational modification (8–10). However, accumulating data have implicated extracellular cyclophilins, including cyclophilin A and CypB, as intercellular mediators in inflammation (8). High levels of extracellular cyclophilins have been detected in several human inflammatory diseases such as severe sepsis, vascular smooth muscle cell disease, atherosclerosis, lupus, and rheumatoid arthritis, playing an important role in regulating the inflammatory process (11).

As a secreted protein with endoplasmic reticulum-directed sequences, CypB can be constitutively secreted by tissue-resident cells such as fibroblasts, chondrocytes, and keratinocytes, especially due to inflammatory stimuli, oxidative stress, and viral infection (12, 13). Circulating CypB may reflect, initiate, and/or aggravate inflammatory responses mainly through recruitment of leukocytes and stimulation of matrix metalloproteinase (MMP) production *via* the extracellular cyclophilin-CD147 pathway, as demonstrated previously (8). Moreover, since inflammatory responses and inflammatory factors play an important role in metabolic complications and the degree of inflammation could reflect the presence and severity of MetS (14), CypB may also act as a valuable biomarker for patients with MetS.

However, the contribution of CypB to MetS has never been established. Therefore, this study aimed to explore a possible relationship between serum CypB levels and MetS as well as each of its components. We also investigated CypB expression in several metabolic organs of both mice with normal diet (ND) and mice with obesity.

## Materials and Methods

### Study Participants

This cross-sectional study was conducted on 503 Chinese subjects continuously recruited from the Department of Cardiology, Shanghai Ruijin Hospital, during their regular health examinations. Subjects with significant concomitant diseases such as proved coronary artery disease, heart failure, infection, autoimmune disease, or cancer were excluded from the analysis. The study protocol was approved by the Institutional Review Board of Ruijin Hospital affiliated to Shanghai Jiao Tong University School of Medicine (Ethics Committee reference number: 2016-019), and was conducted in accordance with the Declaration of Helsinki. Written informed consent was obtained from each participant before data collection per instructions of the review board.

### Data Collection and Measures

Baseline data were collected by trained interviewers *via* face-to-face interviews using a questionnaire; information on medical history, health status, and lifestyle practices were recorded. Standing height, body weight, and waist circumference (WC) were measured with the participants in light indoor clothing and without shoes. WC was measured at a level midway between the lowest lateral border of the ribs and the uppermost lateral iliac crest in the standing position, and body mass index (BMI) was calculated as weight in kilograms divided by square of height in meters. Moreover, blood pressure (systolic and diastolic blood pressure) was measured manually by a calibrated aneroid sphygmomanometer.

For laboratory measurements, fasting peripheral venous EDTA blood samples were collected and centrifuged at 3,000 rpm at 4°C for 15 min. Fasting plasma glucose, total cholesterol, triglycerides, high-density lipoprotein (HDL) cholesterol, low-density lipoprotein (LDL) cholesterol, and other clinical parameters were measured using an automatic analyzer. An oral glucose tolerance test was performed according to the standard protocol to measure the 2-h glucose level. All subjects fasted for at least 8–12 h before the test. After fasting, blood was drawn to establish a fasting glucose level. Next, they quickly drank a sugary (glucose-rich) beverage containing 75 g of carbohydrates. Two hours after the beverage was consumed, blood was redrawn to measure the 2-h glucose levels. CypB levels in the sera were measured in duplicate using a human CypB enzyme-linked immunosorbent assay kit (Cat. No. SEA227Hu, USCN Life Science), according to the manufacturer’s protocol as described previously (15).

### Definition of MetS

Based on the updated National Cholesterol Education Program/Adult Treatment Panel III criteria for Asian-Americans and a more accurate cutoff for WC appropriate for Asian populations in China, MetS was defined as having at least three of the following components (16, 17): WC ≥ 90 cm in men or ≥80 cm in women; triglycerides ≥ 1.7 mmol/L; HDL cholesterol < 1.03 mmol/L in men or <1.30 mmol/L in women; current blood pressure ≥130/85 mmHg or taking antihypertensive medications; or current fasting blood glucose ≥5.6 mmol/L, previously diagnosed type 2 DM, or taking antidiabetic medications including oral antidiabetic agents or insulin.

### Animal Models

Male wild-type (WT; C57BL/6J) mice and genetically obese (ob/ob; C57BL/6J background) mice were purchased at 4 weeks of age and maintained in an animal facility at Ruijin Hospital. The WT mice were randomly divided into two groups: the ND group and the high-fat diet (HFD) group (*n* = 10 mice per group). The ob/ob mice had a spontaneous mutation (Lep^ob^), gaining weight rapidly and reaching twice the normal weight of the WT control mice. Mice in both WT control group and ob/ob group were fed with ND, while the diet-induced obese group received HFD (D12492; Research Diets, New Brunswick, NJ, USA) *ad libitum* for 16 weeks. This HFD contained 60% fat, 20% carbohydrate, and 20% protein. All three mice groups were sacrificed at the age of 20 weeks. The mice were housed under 12/12-h light/dark cycles with free access to food and water. All animal experiments were performed in accordance with the National Institutes of Health Guide for the Care and Use of Laboratory Animals, approved by Ruijin Hospital and Shanghai Jiaotong University School of Medicine Institutional Animal Care and Use Committee.

### Western Blot Analysis

Proteins were separated by sodium dodecyl sulfate polyacrylamide gel electrophoresis (SDS-PAGE; 12.5%) and transferred onto nitrocellulose membranes. After the proteins were transferred, the membranes were blocked in Tris-buffered saline/Tween buffer (TBST; 0.1%, v/v) supplemented with 5% (w/v) nonfat dry milk for 1 h at room temperature, and then probed with primary antibodies to CypB, tumor necrosis factor-α (TNF-α), IL-1β (Abcam, Cambridge, UK), CD68 (Bio-Rad, CA, USA), and β-actin (Beyotime, Shanghai, China) in TBST supplemented with 1% (w/v) bovine serum albumin at 4°C overnight at optimized dilution ratios. Horseradish peroxidase-conjugated secondary antibodies were used after three subsequent washes in TBST, for 80 min at room temperature. Thereafter, enhanced chemiluminescence was performed.

### Real-time Polymerase Chain Reaction (PCR) Analysis

Total RNA was isolated from tissues using the acid-phenol extraction method in the presence of chaotropic salts (TRIzol, Invitrogen, Carlsbad, CA, USA) and subsequent isopropanol-ethanol precipitation. Reverse transcription of mRNA encoding CypB as well as 18S RNA was performed on 2 µg of total RNA using the SuperScript First-Strand Synthesis System (Invitrogen), according to the manufacturer’s protocol. Real-time PCR amplification was performed using the SYBR Green PCR Master Mix (Applied Biosystems, Foster City, CA, USA) and ABI Prism 7500 Sequence Detection System (Applied Biosystems), following the manufacturer’s protocol. Results were calculated using the equation ΔΔCT. Primer sets were as follows: 18S RNA: former 5′→3′ TGGTTGCAAAGCTGAAACTTAAAG, reserve 5′→3′ AGTCAAATTAAGCCGCAGGC. CypB: former 5′→3′ TATGAAGGTGCTCTTCGCCG, reserve 5′→3′ AGTATACCTTGACTGTGACTTTAGG. The primer sequences of 18S RNA can be used for both humans and mice, while the CypB sequences are only for mice.

### Statistical Analyses

All data management and statistical analyses were conducted using SPSS 22.0. For continuous variables, data were summarized as mean ± SD, and for categorical variables, data were shown as numbers (percentage) in each group. Variables with a skewed distribution were log transformed to approximate normality before analysis.

The differences between groups were compared using the *t*-test and chi-square test as appropriate. Correlation coefficients between CypB and metabolic features were calculated by Pearson’s correlation analysis. Subsequently, univariate and multivariate logistic regression models were used to evaluate the association between serum CypB level and the risk for MetS, using both CypB and log-transformed CypB as continuous variables analyzed per SD. The relationship between MetS and serum CypB level was also explored in subgroup analysis, conducted within strata of age (≥60/<60 years old) and gender (men/women). All statistical tests were two-sided, and a *P*-value <0.05 was considered statistically significant.

## Results

### Baseline Characteristics

Among all enrolled subjects in this cross-sectional study, 211 were diagnosed with MetS and 292 belonged to the non-MetS group, including 133 healthy controls and 159 high-risk subjects differentiated by one to two MetS components, insufficient for a MetS diagnosis. As summarized in Table 1, the subjects of all groups were of almost the same age, and the gender distribution was also almost equal; however, those with MetS seemed to be more likely to fall into unhealthy habits such as smoking and alcohol use. The comparisons of five MetS-defining components, namely, increased WC, hypertension, elevated fasting blood glucose, elevated triglycerides, and low HDL cholesterol, and several clinical parameters as well as medication use among the healthy group, high-risk group, and MetS subjects are all listed in Table 1. These clinical parameters, including blood pressure, lipid profile, and glucose levels, were also significantly higher in the high-risk group compared with the healthy controls. Moreover, inflammatory markers such as C-reactive protein (CRP), TNF-α, and white blood cell (WBC) levels were also significantly higher in the MetS group, indicating a more severe inflammatory condition.

**Table 1:** Baseline characteristics of subjects with and without MetS.

	Non-MetS, healthy group (*n* = 133)	Non-MetS, high-risk group (*n* = 159)	MetS (*n* = 211)	*P*-value
Age, years	57.29 ± 10.11	59.59 ± 9.12	59.21 ± 8.76	0.077
Male, gender	63 (47.4)	76 (47.8)	117 (55.5)	0.221
Smoker	24 (18)	31 (19.5)	59 (28.0)	0.052
Alcohol use	16 (12.0)	20 (12.6)	40 (19.0)	0.122
Family history	9 (6.8)	16 (10.1)	22 (10.4)	0.489
BMI (kg/m^2^)	21.66 ± 2.20	23.14 ± 2.94	26.42 ± 3.04	<0.001
Waist circumference (cm)	75.06 ± 7.23	80.25 ± 11.01	89.07 ± 11.42	<0.001
Systolic blood pressure (mmHg)	120.24 ± 11.92	128.32 ± 15.83	137.96 ± 19.00	<0.001
Diastolic blood pressure (mmHg)	72.55 ± 9.54	74.25 ± 11.25	79.27 ± 11.38	<0.001
**Medical history**				
Hypertension	0 (0)	67 (42.1)	145 (68.7)	< 0.001
Diabetes mellitus	0 (0)	12 (7.5)	51 (24.2)	<0.001
Dyslipidemia	0 (0)	4 (2.5)		
**Biochemical measurements**				
WBC (×10^9^)	5.58 ± 1.52	6.10 ± 1.92	6.72 ± 1.75	<0.001
Hemoglobin (g/L)	132.20 ± 13.87	130.79 ± 16.60	135.45 ± 16.11	0.014
Platelet (×10^9^)	180.05 ± 55.13	188.79 ± 64.17	193.81 ± 56.39	0.107
Fasting glucose (mmol/L)	4.77 ± 0.50	5.01 ± 1.01	5.60 ± 1.24	<0.001
HbA1c (%)	5.56 ± 0.38	5.75 ± 0.54	6.27 ± 1.09	<0.001
ALT (IU/L)	19.54 ± 11.81	22.91 ± 16.83	27.72 ± 16.58	<0.001
eGFR_MDRD_ (ml/min/1.73 m^2^)	85.57 ± 15.84	80.54 ± 16.71	80.69 ± 20.82	0.029
Uric acid (μmol/L)	297.23 ± 69.97	315.28 ± 96.31	359.13 ± 98.24	<0.001
Total cholesterol (mmol/L)	4.38 ± 0.87	4.32 ± 0.95	4.36 ± 1.11	0.850
Triglyceride (mmol/L)	1.06 ± 0.38	1.26 ± 0.54	2.13 ± 1.26	<0.001
LDL-C (mmol/L)	2.56 ± 0.73	2.55 ± 0.75	2.59 ± 0.89	0.880
HDL-C (mmol/L)	1.35 ± 0.34	1.29 ± 0.30	0.99 ± 0.23	<0.001
CRP (mg/L)	1.57 ± 1.69	3.56 ± 3.44	4.48 ± 3.51	<0.001
TNF-α (pg/mL)	28.00 ± 13.67	31.15 ± 13.02	32.06 ± 14.66	0.028
CypB (ng/mL)	124.26 ± 47.83	168.38 ± 65.01	193.80 ± 83.22	<0.001
**Medications**				
Statin	0 (0)	60 (37.7)	101 (47.9)	<0.001
ACEI/ARB	0 (0)	58 (36.5)	103 (48.8)	<0.001
Hypoglycemic drugs	0 (0)	9 (5.7)	40 (19.0)	<0.001
β-blocker	16 (12.0)	94 (59.1)	143 (67.8)	<0.001
CCB	2 (1.5)	31 (19.5)	64 (30.3)	<0.001
Aspirin	49 (36.8)	82 (51.6)	128 (60.7)	<0.001

*Data are presented as mean ± SD or *n* (%)*.

*MetS, metabolic syndrome; BMI, body mass index; WBC, white blood cell; HbA1c, glycated hemoglobin; ALT, alanine aminotransferase; eGFR, estimated glomerular filtration rate; LDL-C, low-density lipoprotein cholesterol; HDL-C, high-density lipoprotein cholesterol; CRP, C-reactive protein; TNF-α, tumor necrosis factor-α; CypB, cyclophilin B; ACEI, angiotensin-converting enzyme inhibitors; ARB, angiotensin receptor blockers; CCB, calcium channel blockers*.

### Serum CypB Level Was Associated with the Prevalence and Severity of MetS

**Figure 1:**
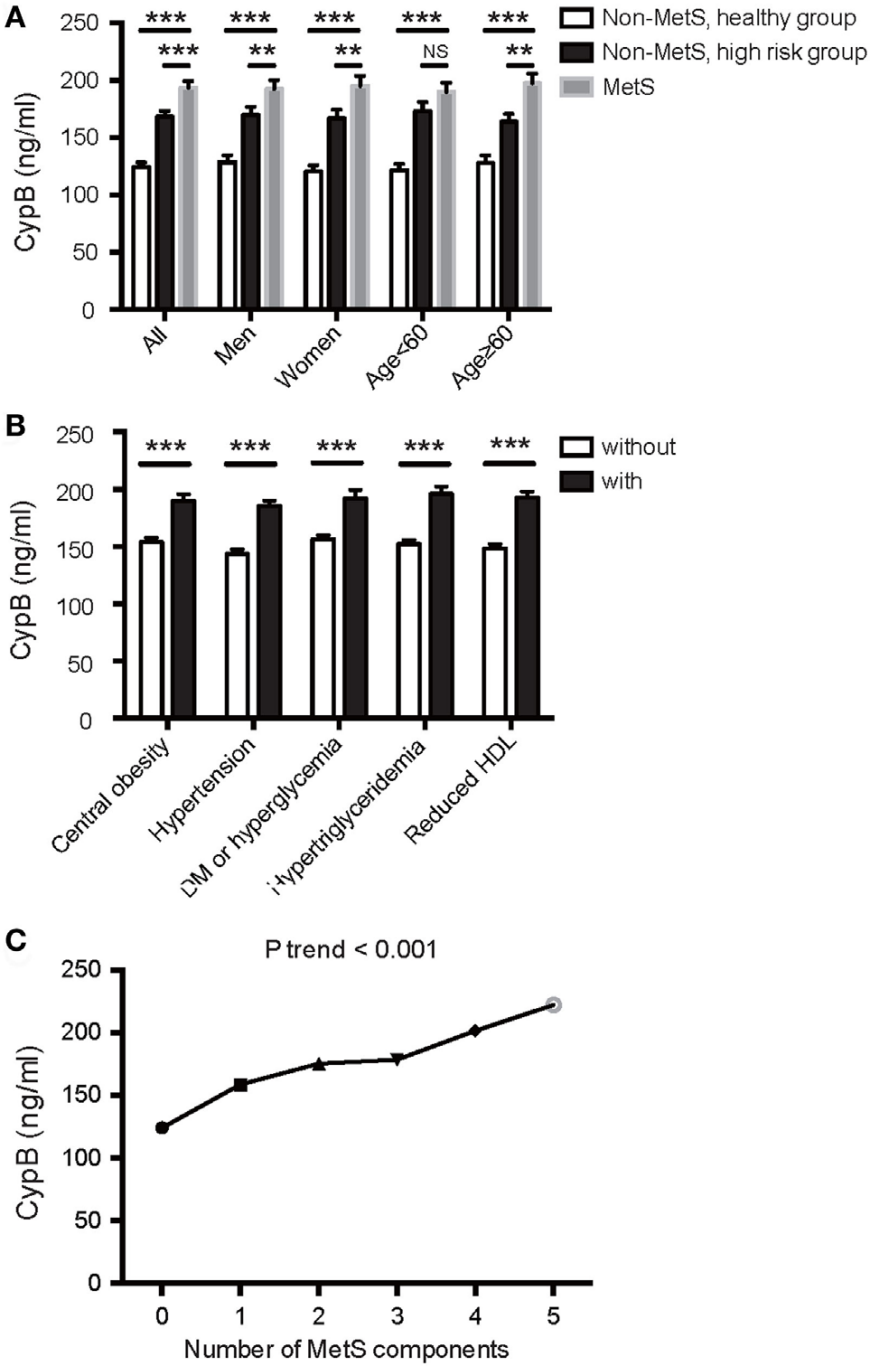
Association of serum cyclophilin B (CypB) levels with metabolic syndrome (MetS) and its components. **(A)** Comparison of serum CypB levels in all subjects comprising the non-MetS healthy group, non-MetS high-risk group, and MetS group. Stratified analyses were conducted by age (<60/≥60 years) and gender (men/women) for the association between MetS and CypB. **(B)** Serum CypB levels were compared between patients grouped by individual MetS components: central obesity, hypertension, DM or hyperglycemia, hypertriglyceridemia, and reduced HDL. **(C)** Correlation analysis between CypB levels and the number of MetS components. NS, not significant; *P < 0.05, **P < 0.01, ***P < 0.001.

Serum CypB level was significantly higher in MetS subjects than in both groups of non-MetS subjects (193.80 ± 83.22 vs. 168.38 ± 65.01 vs. 124.26 ± 47.83 ng/mL, *P* < 0.001) and was also higher in the high-risk group compared with the healthy controls (Figure 1A). In this study, when the subjects were divided according to each of the components described above, serum CypB level was significantly higher in subjects with hypertension, central obesity, DM or elevated fasting blood glucose, elevated triglycerides, or reduced HDL cholesterol than in those without (Figure 1B).

Simple linear regression analysis revealed a significant positive correlation between levels of log CypB and triglycerides, fasting blood glucose, 2-h glucose, hemoglobin A1c (HbA1c), blood pressure, BMI, and WC. Furthermore, participants with a higher serum CypB level had a lower HDL cholesterol level (Table 2). All these findings confirmed an association between CypB and obesity, together with glucose and the lipid metabolic process. Serum CypB level increased gradually with increasing number of MetS components, from the subjects with none of the components (124.26 ± 47.83 ng/mL) to those with all five components (222.42 ± 114.41 ng/mL) (Figure 1C), thus confirming the internal conformance of the abovementioned findings (*P* trend < 0.001). Moreover, CypB was remarkably associated with the number of MetS components (*r* = 0.404, *P* < 0.001) (Table 2), indicating that a higher serum CypB level reveals more severe MetS.

**Table 2:** Correlations between log CypB and metabolic parameters.

	*r*	*P*
Age	−0.014	0.758
BMI	0.208	<0.001
Waist circumference	0.194	<0.001
Systolic blood pressure	0.122	0.006
Diastolic blood pressure	0.085	0.057
Fasting glucose	0.160	<0.001
2 h glucose	0.224	<0.001
HbA1c	0.201	<0.001
Triglyceride	0.260	<0.001
Total cholesterol	0.018	0.702
HDL-C	−0.202	<0.001
LDL-C	0.009	0.850
MetS score	0.404	<0.001
Log WBC	0.297	<0.001
Log CRP	0.296	<0.001
Log TNF-α	−0.020	0.648

*BMI, body mass index; HbA1c, glycated hemoglobin; TC, total cholesterol; HDL-C, high-density lipoprotein-cholesterol; LDL-C, low-density lipoprotein-cholesterol; WBC, white blood cells; CRP, C-reactive protein; TNF, tumor necrosis factor*.

Meanwhile, serum CypB levels were found to be remarkably associated with WBC and CRP levels (Table 2), indicating an association between CypB and the general inflammatory condition. However, CypB was not significantly correlated with TNF-α levels in our subjects, suggesting the different pathways through which they affected the inflammatory status.

Next, we explored the independent association between serum CypB level and the risk for prevalent MetS, taking both CypB and log-transformed CypB as continuous variables (Table 3). The association remained significant when unadjusted [OR, 1.976 (1.600–2.439); *P* < 0.001], adjusted for age and gender [OR, 1.968 (1,594–2.540); *P* < 0.001], and further adjusted in the full model [OR, 1.506 (1.080–2.101); *P* = 0.016], including age, gender, alcohol use, smoking, BMI, current blood pressure, HbA1c, total cholesterol, LDL cholesterol, estimated glomerular filtration rate, statin use, antihypertension medication, anti-DM medication, aspirin, WBCs, CRP, and TNF-α.

**Table 3:** Uni- and multivariable logistic regression analysis for MetS in all subjects and subjects divided by age and gender.

	Unadjusted OR	*P*-value	Adjusted OR for model 1	*P*-value	Adjusted OR for model 2	*P*-value
**All subjects**						
Log Cypb per SD	2.003 (1.632–2.457)	<0.001	1.993 (1.624–2.446)	<0.001	1.456 (1.061–1.998)	0.020
Cypb per SD	1.976 (1.600–2.439)	<0.001	1.968 (1.594–2.430)	<0.001	1.506 (1.080–2.101)	0.016
**Age < 60**						
Log Cypb per SD	1.992 (1.498–2.648)	<0.001	1.989 (1.489–2.655)	<0.001	1.342 (0.820–2.196)	0.242
Cypb per SD	1.912 (1.432–2.553)	<0.001	1.906 (1.420–2.559)	<0.001	1.307 (0.799–2.140)	0.287
**Age ≥ 60**						
Log Cypb per SD	2.014 (1.501–2.702)	<0.001	1.918 (1.403–2.610)	<0.001	1.513 (0.978–2.342)	0.063
Cypb per SD	2.048 (1.505–2.787)	<0.001	1.953 (1.407–2.696)	<0.001	1.601 (1.015–2.527)	0.043
**Men**						
Log Cypb per SD	1.898 (1.429–2.522)	<0.001	1.710 (1.236–2.340)	0.002	1.449 (0.876–2.397)	0.149
Cypb per SD	1.899 (1.413–2.552)	<0.001	1.709 (1.218–2.368)	0.001	1.451 (0.840–2.506)	0.145
**Women**						
Log Cypb per SD	2.103 (1.565–2.825)	<0.001	1.908 (1.468–2.734)	<0.001	1.869 (1.177–2.967)	0.008
Cypb per SD	2.046 (1.514–2.766)	<0.001	1.951 (1.416–2.674)	<0.001	1.784 (1.036–3.070)	0.037

*Model 1: adjusted for age and gender*.

*Model 2: adjusted for age, gender, alcohol use, smoke, BMI, current blood pressure, HbA1c, TC, LDL, eGFR, statin use, antihypertension medication, anti DM medication, aspirin, WBC, CRP, and TNF-α*.

*BMI: <18.5 = 1, 18.5–25 = 2, 25–28 = 3, 28–32 = 4, and ≥32 = 5*.

*Current blood pressure: <120 and <80 = 1, 120–139/80–89 = 2, 140–159/90–99 = 3, 160–179/100–109 = 4, ≥180/≥110 = 5*.

*Age: per 5 years*.

*Other continuous variables were entered per 1 SD*.

Serum CypB level in both MetS and non-MetS subjects remained significantly different regardless of gender, while its level was significantly higher in the MetS group than in the non-MetS high-risk group in older subjects (aged ≥ 60 years), but not in those <60 years old (Figure 1A). Stratified analyses were conducted by age and gender for the association between MetS and CypB (Table 3). We demonstrated that older subjects with a higher CypB level were more likely to have MetS. In addition, the risk for MetS was higher and more significant in women compared with men. In conclusion, serum CypB levels were independently associated with the risk for MetS in all subjects analyzed, especially in women and in subjects older than 60 years.

### Increased CypB Expression in the Liver and Adipose Tissue in HFD-Induced Obese Mice and ob/ob Mice

**Figure 2:**
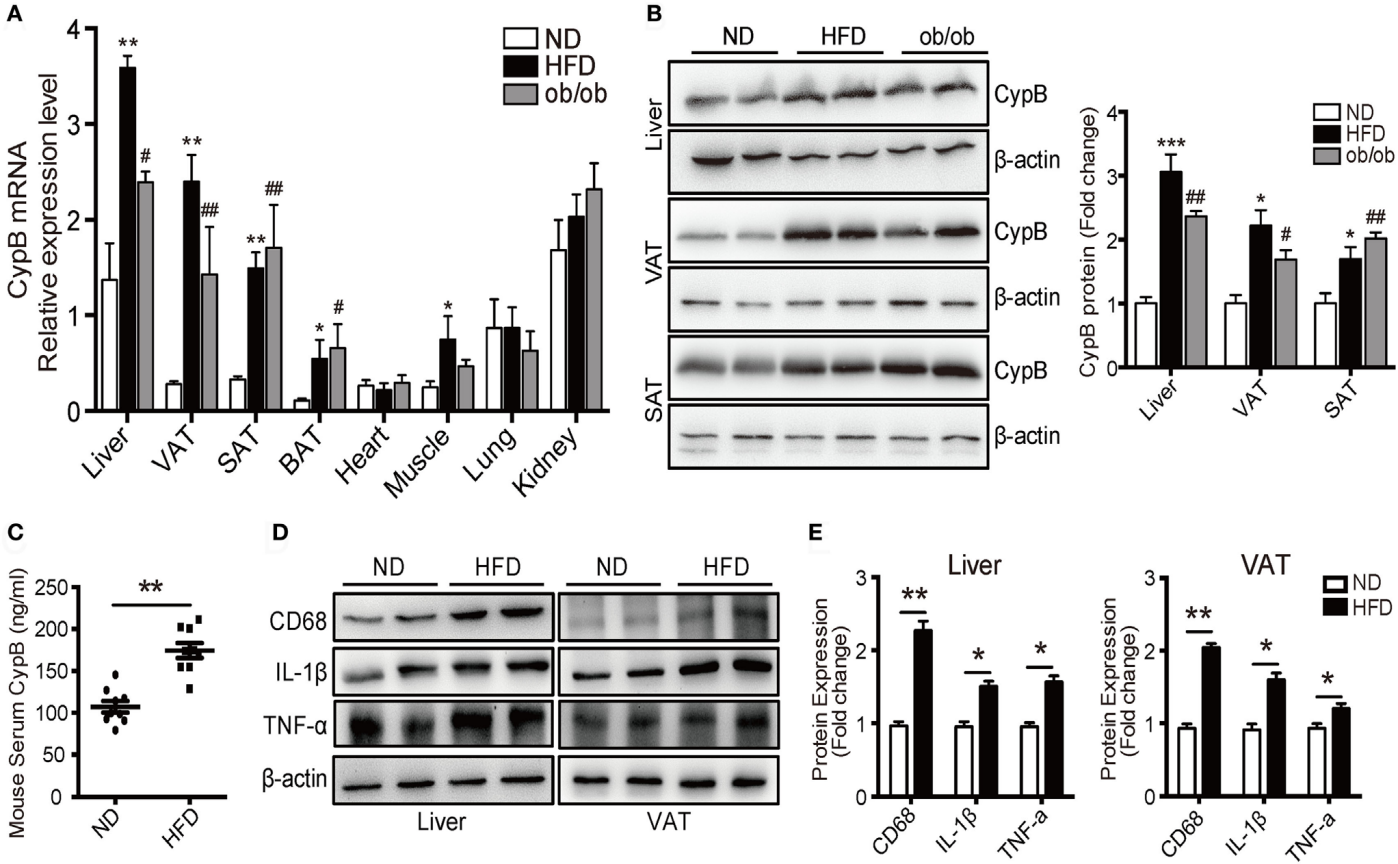
Increased cyclophilin B (CypB) expression was observed in obese mice fed with high-fat diet (HFD) and in genetically obese (ob/ob) mice. **(A)** The expression profile of CypB was determined by real-time PCR in different organs and tissues from mice fed with normal diet (ND) or HFD and from ob/ob mice. **(B)** The protein levels of CypB were examined by Western blot analysis in visceral (VAT) and subcutaneous adipose tissue (SAT) and the liver from ND mice, HFD-induced obese mice, and ob/ob mice. **(C)** Serum CypB levels were compared between ND mice and HFD mice. **(D,E)** Representative Western blot analyses and summary data showing the protein expression of CD68, IL-1β, and TNF-α in the liver and VAT from both ND mice and HFD mice (*n* = 6 mice per group). *P < 0.05, **P < 0.01, ***P < 0.001 vs. the ND group; ^#^*P* < 0.05, ^##^*P* < 0.01, ^###^*P* < 0.001 vs. the ND group.

Using real-time PCR and Western blot analysis, we examined the expression profile of CypB in different organs and tissues from mice fed with ND or HFD, and from ob/ob mice. At baseline, mRNA levels of CypB were highly expressed in the liver, lung, and kidney but lowly expressed in visceral (VAT), subcutaneous (SAT), and brown adipose tissue (BAT) and heart and skeletal muscles in ND mice (Figure 2A). However, when the mice were challenged to HFD or genetic deficiency of leptin (ob/ob), CypB expression levels were dramatically enhanced in metabolic organs such as the liver and in VAT, SAT, BAT, and skeletal muscles, compared with ND mice, although CypB expression in the heart, lung, and kidney did not significantly change. In line with these findings, CypB protein expression levels were also increased in the liver, VAT, and SAT from HFD and ob/ob mice, compared with ND mice (Figure 2B). Moreover, serum levels of secreted CypB were also higher in HFD mice than in ND mice (Figure 2C).

Consistent with the results of human studies, the expression of inflammatory markers including IL-1β and TNF-α as well as the macrophage marker CD68 was significantly higher in the liver and VAT of HFD mice compared with ND ones (Figures 2D,E). These results indicate that CypB expression correlates with the accumulation of immune cells known to contribute to metabolic complications, especially in metabolic organs.

## Discussion

### Major Findings

In this cross-sectional observational study, significant associations were detected between serum CypB level and the occurrence and severity of MetS as well as each of its components. Notably, multivariate logistic regression analysis further demonstrated that CypB was an independent risk factor for the presence of MetS, especially in women and in subjects older than 60 years. Moreover, the expression levels of CypB were only increased in murine metabolic organs such as the liver and adipose tissues in response to HFD and genetic deficiency of leptin. Meanwhile, serum CypB levels were elevated accordingly, showing similarity to human data. To our knowledge, this study is the first to consider serum CypB as a novel biomarker for MetS in humans and detect its expression pattern in murine metabolic organs. Our results clearly indicate the association of CypB and MetS in both humans and mice, and CypB may participate in MetS by mediating the inflammatory process and lipid metabolism; however, the underlying mechanisms remain incompletely understood.

### MetS Promotes the Release of CypB

Similar to cyclophilin A (CypA), extracellular CypB has been demonstrated to increase in response to inflammatory stimuli and oxidative stress (18), and serves as a serum biomarker in several inflammatory diseases, such as acute lung inflammation, rheumatoid arthritis, Sjögren’s syndrome, and cardiovascular disease (8). MetS is characterized by complex interactions between inflammation and a dysregulated metabolism. Furthermore, the fact that systemic inflammation may critically coexist with the presence of MetS was verified in several clinical studies (19, 20). Metabolically triggered inflammation, also known as meta-inflammation, is a special inflammatory condition principally triggered by nutrients and metabolic surplus, but engages a set of cytokines and signaling pathways similar to those involved in a classical inflammation (21). Thus, MetS, including obesity, insulin resistance, and lipid metabolism disorders, is crucially linked to the inflammatory process and immune response. Taking together the facts that MetS is a chronic inflammatory condition of some kind (22) and that inflammatory stimuli trigger the expression and release of CypB (18), serum CypB level is reasonably increased in patients with MetS.

On the other hand, since obesity increases both the size and the number of adipocytes, resulting in increased macrophage infiltration and proinflammatory status, the inflammatory state is more severe in obese subjects than in lean subjects (23). This may, in part, explain why CypB is overexpressed in murine models with obesity. Moreover, metabolic organs such as the liver, VAT, and SAT may majorly promote the release of CypB since in this study, CypB expression was only enhanced in these organs in HFD and ob/ob mice.

However, it is still unclear which cells, adipocytes or infiltrated macrophages, are the main producers of CypB in obesity and which signaling pathways are responsible for CypB expression and secretion. Thus, further studies are needed to elucidate these unsolved questions.

### CypB Affects MetS *via* Its Proinflammatory Function

Extracellular CypB participates in several inflammatory conditions and diseases as a regulator of inflammation, as mentioned above (8, 12). However, evidence supporting the potential role of CypB in the development of MetS and its related diseases is still limited. As a family member of cyclophilins, with a similar structure and function with CypB, extracellular CypA has been demonstrated to initiate a cascade of inflammatory cardiovascular processes including vascular remodeling, atherosclerosis, myocardial infarction, and inflammatory and noninflammatory cardiomyopathies, and to also relate to MetS, mainly through binding to its extracellular receptor—CD147 (24). CD147, also named extracellular MMP inducer, has been identified as a key regulator for transmitting cellular signals mediating metalloproteinase activities, participating in the inflammation process and oxidative stress (25, 26). Previous studies in both human and animal models have suggested that CD147 plays an important part in the development of MetS and its related cardiovascular diseases (27). As a ligand of CD147 (28), CypB may likewise affect such diseases through its binding with CD147 to some extent.

Furthermore, this study revealed that CypB is significantly associated with WBCs, which have been found to be positively correlated with MetS as a marker of inflammation in both young adults and the elderly (29, 30). To support this, accumulating data have indicated a key role for secreted CypB as a regulator of inflammation (28, 31), contributing to innate immune reaction as a supplement of the chemokine group (12). As evidenced by *in vitro* experiments, extracellular CypB induces chemotaxis and integrin-mediated adhesion of T cells to the extracellular matrix by way of interaction with two classes of receptors—syndecan-1 and CD147 (18, 32). By binding with CD147, CypB triggers chemotaxis of polymorphonuclear leukocytes, monocytes, and T lymphocytes, contributing to the guidance of leukocyte populations to inflammatory sites and aggregating the inflammation process (31, 33).

On the other hand, by activating CD147, CypB has been reported to trigger a peculiar ERK-MAPK signaling pathway and induce the release of MMPs (8, 18), which could participate in the process of degradation of extracellular matrix, initiating, or exacerbating the pathogenesis of MetS and related diseases (34).

### CypB and Lipid Metabolism

Metabolic organs including the liver and adipose tissue are of vital importance in the initiation and development of metabolic diseases, especially in the context of obesity and type 2 DM (35). As demonstrated in our study, the expression level of CypB was only enhanced in the liver, VAT, and SAT, rather than in the lung, kidney, and heart in murine models of obesity, suggesting that CypB may contribute to the effect of these organs and tissue, leading to metabolic disorder.

Moreover, serum CypB levels were more significantly associated with hypertriglyceridemia and reduced HDL cholesterol level than with hypertension and DM or hyperglycemia, among all components of MetS, further suggesting the relationship between CypB and lipid metabolism. The correlation between CypB and triglycerides or HDL cholesterol was also quite remarkable. In the murine model of obesity, the expression level of inflammatory markers including IL-1β and TNF-α as well as the macrophage marker CD68 was significantly enhanced in metabolic organs, together with CypB. One of the underlying mechanisms is that CypB overexpression in adipose tissue can trigger chemotaxis of immune cells such as macrophages, neutrophils, and eosinophils (31), which further aggravates inflammation or lipid metabolism as discussed above. Nonetheless, the possible role of CypB on adipogenesis or the course of lipid metabolism needs to be elucidated in future studies. Meanwhile, recent studies demonstrated that CypA was a novel adipogenic factor important in obesity, and overexpression of CypA could directly affect adipogenesis and induce obesity (36). Considering that CypB expression was increased in the adipose tissue of obese mouse models in this study, coupled with the similar structure and function of CypA and CypB, we reasonably suppose that CypB may also directly act on adipose tissue and lipid metabolism; however, this needs to be further explored.

### Study Limitations

Nevertheless, this study has several limitations.

First, since the sample size was limited in this study, further studies on a larger group derived from other populations with different genetic and environmental backgrounds are warranted to confirm our findings and better understand the complicated mechanism of MetS.

Second, the study’s cross-sectional design implicated that no causal inference can be discerned. Therefore, the correlation of CypB with the risk for MetS needs to be validated in larger study cohorts to further determine the predictive or prognostic value of CypB.

Additionally, serum CypB level could possibly be affected by several unmeasured confounders or unknown conditions, such as specific drugs that influence the secretion or metabolism of CypB as well as the inflammation state or hormones that were able to affect vascular function and glucose or lipid metabolism, taking part in the pathogenesis of MetS and its components. Although we adjusted for some confounding factors in this study, some variables still need to be identified and analyzed.

## Conclusion

This study determined that serum CypB levels were significantly and independently associated with the presence and severity of MetS. Moreover, CypB was found to be mainly expressed in metabolic organs such as the liver and adipose tissue, and the expression of CypB was significantly increased in obese murine models, indicating that CypB could be used as a novel biomarker and clinical predictor of MetS.

## Ethics Statement

This cross-sectional study was conducted on 503 Chinese subjects continuously recruited from the Department of Cardiology, Shanghai Ruijin Hospital, during their regular health examinations. Subjects with significant concomitant diseases such as proved coronary artery disease, heart failure, infection, autoimmune disease, or cancer were excluded from the analysis. The study protocol was approved by the Institutional Review Board of Ruijin Hospital affiliated to Shanghai Jiao Tong University School of Medicine (Ethics Committee reference number: 2016-019) and was conducted in accordance with the Declaration of Helsinki. Written informed consent was obtained from each participant before data collection per instructions of the review board.

## Author Contributions

All authors made substantial contributions to the work and preparation of the article. HZ, QF, and XY contributed to all stages, including the conception and design of the study as well as the acquisition, analysis, and interpretation of data. HZ and QF contributed to the drafting of the article, together with XY in the revising and final approval of the version to be submitted. HX, LL, RT, JH, FW, RX, QC, WS, and RZ were integral to the design of the study as well as data collection. All authors have approved the final article.

## Conflict of Interest Statement

The authors declare that the research was conducted in the absence of any commercial or financial relationships that could be construed as a potential conflict of interest.
